# Predicting Mutational Status of Driver and Suppressor Genes Directly from Histopathology With Deep Learning: A Systematic Study Across 23 Solid Tumor Types

**DOI:** 10.3389/fgene.2021.806386

**Published:** 2022-02-16

**Authors:** Chiara Maria Lavinia Loeffler, Nadine T. Gaisa, Hannah Sophie Muti, Marko van Treeck, Amelie Echle, Narmin Ghaffari Laleh, Christian Trautwein, Lara R. Heij, Heike I. Grabsch, Nadina Ortiz Bruechle, Jakob Nikolas Kather

**Affiliations:** ^1^ Department of Medicine III, University Hospital RWTH Aachen, Aachen, Germany; ^2^ Center for Integrated Oncology Aachen Bonn Cologne Duesseldorf (CIO ABCD), Aachen, Germany; ^3^ Institute of Pathology, University Hospital RWTH Aachen, Aachen, Germany; ^4^ Department of Surgery and Transplantation, University Hospital RWTH Aachen, Aachen, Germany; ^5^ NUTRIM School of Nutrition and Translational Research in Metabolism, Maastricht University, Maastricht, Netherlands; ^6^ Department of Pathology, GROW School for Oncology and Reproduction, Maastricht University Medical Center+, Maastricht, Netherlands; ^7^ Pathology and Data Analytics, Leeds Institute of Medical Research at St James’s, University of Leeds, Leeds, United Kingdom; ^8^ Medical Oncology, National Center for Tumor Diseases (NCT), University Hospital Heidelberg, Heidelberg, Germany

**Keywords:** deep learning, artificail intelligence (AI), cancer pathway, cancer pathway genes, genetic, TCGA

## Abstract

In the last four years, advances in Deep Learning technology have enabled the inference of selected mutational alterations directly from routine histopathology slides. In particular, recent studies have shown that genetic changes in clinically relevant driver genes are reflected in the histological phenotype of solid tumors and can be inferred by analysing routine Haematoxylin and Eosin (H&E) stained tissue sections with Deep Learning. However, these studies mostly focused on selected individual genes in selected tumor types. In addition, genetic changes in solid tumors primarily act by changing signaling pathways that regulate cell behaviour. In this study, we hypothesized that Deep Learning networks can be trained to directly predict alterations of genes and pathways across a spectrum of solid tumors. We manually outlined tumor tissue in H&E-stained tissue sections from 7,829 patients with 23 different tumor types from The Cancer Genome Atlas. We then trained convolutional neural networks in an end-to-end way to detect alterations in the most clinically relevant pathways or genes, directly from histology images. Using this automatic approach, we found that alterations in 12 out of 14 clinically relevant pathways and numerous single gene alterations appear to be detectable in tissue sections, many of which have not been reported before. Interestingly, we show that the prediction performance for single gene alterations is better than that for pathway alterations. Collectively, these data demonstrate the predictability of genetic alterations directly from routine cancer histology images and show that individual genes leave a stronger morphological signature than genetic pathways.

## Introduction

**FIGURE 1:**
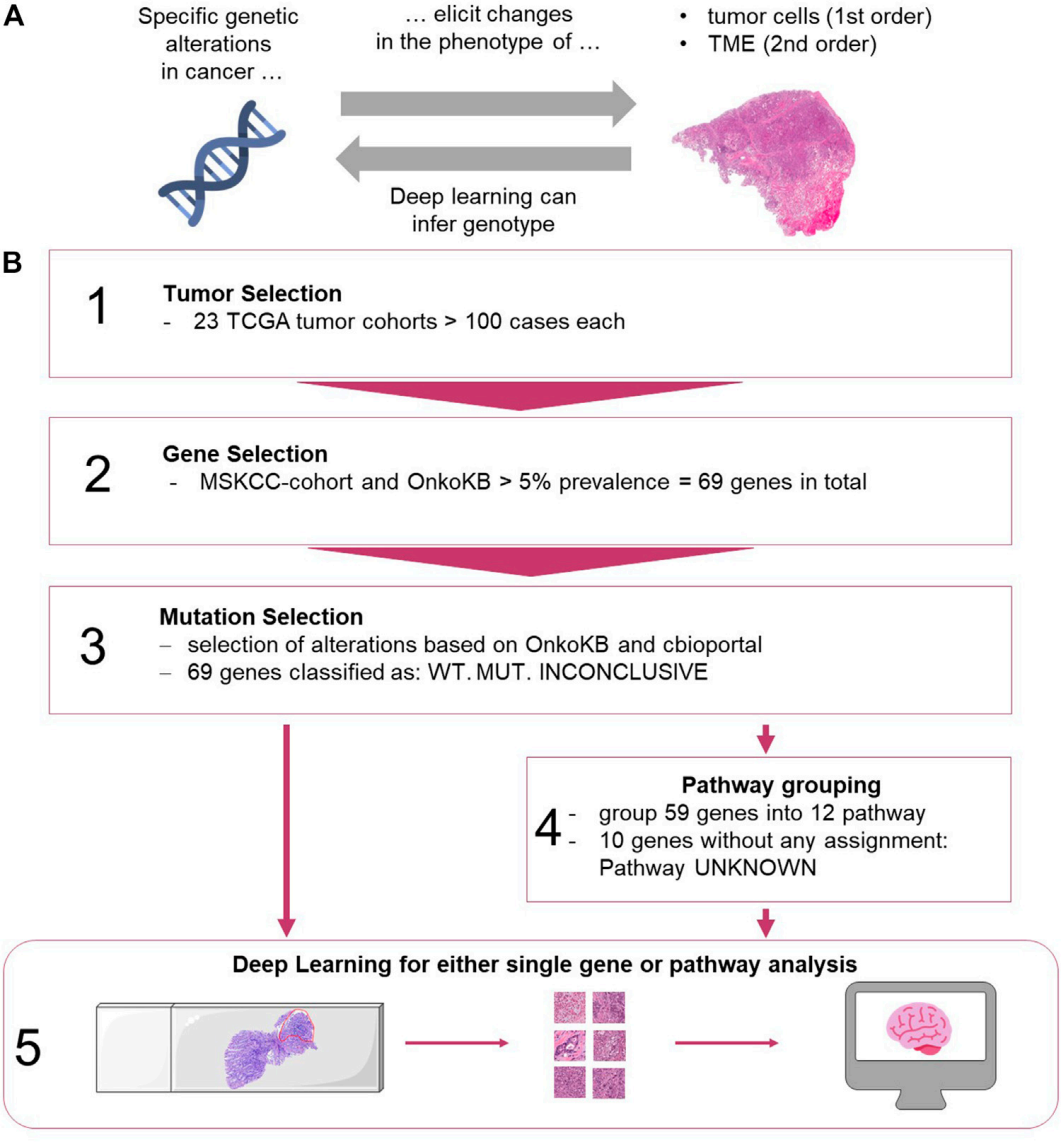
**(A)** Biological hypothesis of this study. TME: tumor microenvironment. **(B)** Workflow for selection of data and Deep Learning methods. **(1)** Tumors from TCGA were analyzed. **(2)** Genes were selected based on the MSKCC cohort and OnkoKB platforms. **(3)** Alterations were grouped based on different sources. **(4)** Genes were grouped into pathways (see Supplementary Table S1). **(5)** Processing of images and training of the network for genes alone and grouped into pathways. (images from https://smart.servier.com, and Twitter Twemoji under a CC-BY license). TME: tumor microenvironment, TCGA: The Cancer Genome Atlas, MSKCC, OnkoKB, WT: wild type, MUT: mutation present.

Genetic changes can influence the cell and tissue morphology of solid tumors (Figure 1A). This morphology can be observed in routine histopathology images which are available for almost every patient with any solid tumor. Routinely, histopathologists review H&E stained tissue sections to establish a diagnosis, stage a disease etc. Due to recent advances in computer vision, automatic image analysis can extract subtle features from digital tissue sections which seem to be elusive to the human eye (Echle et al., 2020b). In particular, Deep Learning (DL), an artificial intelligence method, has been used to analyze histology images (Kather and Calderaro, 2020) and multiple studies demonstrated that Deep Learning can link morphological changes in cancer histology images to specific genetic alterations. Early studies in the field predicted clinically relevant genetic mutations in lung cancer (Coudray et al., 2018), colorectal cancer (Kather et al., 2019), breast cancer (Naik et al., 2020) and other tumor types from histological whole slide images. More recently, multiple studies suggested that many genetic alterations are predictable from routine histology alone across different tumor types (Fu et al., 2020; Kather et al., 2020; Schmauch et al., 2020; Loeffler et al., 2021; Muti et al., 2021). Previous studies focused on predicting single gene alterations. However, it is well known that certain gene products act together in functional pathways and mutations (MUT) of different genes of the same pathway may have a similar effect such as pathway activation (Ben-Hamo et al., 2020). To understand the effect of genetic alterations on tumor biology, potential genetic alterations need to be considered in the context of their functional significance in the affected pathway. For example, it has been shown that both, *PTEN* loss and *PIK3CA* mutation can lead to the activation of the *PI3K* or *MAPK* pathway in cancer of the breast, colorectum, stomach or lung (Dhillon et al., 2007; Jiang et al., 2020). This phenomenon, can be of therapeutic relevance, as targeted therapies may not only affect one specific gene, but also affect other downstream genes. Instead of focusing on a single gene, in some cases it might even be sufficient to identify pathway activation or inhibition to predict treatment response or failure (Schumacher et al., 2019; Ben-Hamo et al., 2020).

We hypothesized that alteration of a particular signaling pathway leads to histomorphological changes which can be predicted from routinely stained pathology slides using Deep Learning technology.

The aim of the current study was to systematically compare the predictability of an “overall altered signaling pathway” to a “single altered gene” of the same pathway. To this end, we analyzed the 69 most frequently mutated genes in 23 cancer types, representing 12 oncogenic pathways, and trained end-to-end Deep Learning networks to predict single gene mutations or signaling pathway alterations. Furthermore, we aimed to extend the evaluation of Deep Learning-based detection of genetic alterations from FFPE slides to a broad range of tumor types, beyond the findings of previous studies which were limited in their selection of genetic alterations (Kather et al., 2020).

## Materials and Methods

### Ethics Statement

All experiments were conducted in accordance with the Declaration of Helsinki and the International Ethical Guidelines for Biomedical Research Involving Human Subjects. Anonymized scanned whole slide images were retrieved from The Cancer Genome Atlas (TCGA) project through the Genomics Data Commons (GDC) Portal (https://portal.gdc.cancer.gov/).

### Patient Cohorts

Digitized hematoxylin/Eosin (H and E) stained slides and molecular data from all solid tumor types with more than 100 cases in the GDC database were included in the analysis: bladder urothelial carcinoma [BLCA, *n* = 332 patients, (Robertson et al., 2017)], breast cancer [BRCA, *n* = 977, (Cancer Genome Atlas Network, 2012b)], cervical cancer [CESC, *n* = 253, (Cancer Genome Atlas Research Network et al., 2017a)], colorectal cancer [COAD and READ, merged as CRC, *n* = 499, (Cancer Genome Atlas Network, 2012a)], esophageal cancer [ESCA, *n* = 153, (Cancer Genome Atlas Research Network et al., 2017b)], glioblastoma [GBM, *n* = 200, (Brennan et al., 2013)], head and neck squamous cell carcinoma [HNSC, *n* = 429, (Cancer Genome Atlas Network, 2015a)], clear cell renal cell carcinoma [KIRC, *n* = 376, (Cancer Genome Atlas Research Network, 2013)], papillary renal cell carcinoma [KIRP, *n* = 240, (Cancer Genome Atlas Research Network et al., 2016)], low grade glioma [LGG, *n* = 480, (Cancer Genome Atlas Research Network et al., 2015)], hepatocellular carcinoma [LIHC, *n* = 352, (Cancer Genome Atlas Research Network, 2017b)], lung adenocarcinoma [LUAD, *n* = 457, (Cancer Genome Atlas Research Network, 2014b)], lung squamous cell carcinoma [LUSC, *n* = 410, (Cancer Genome Atlas Research Network, 2012)], ovarian cancer [OV, *n* = 97 after exclusion of non-analyzable samples; nine patients were excluded during analysis, (Cancer Genome Atlas Research Network, 2011)], pancreatic cancer [PAAD, *n* = 166, (Cancer Genome Atlas Research Network, 2017c)], pheochromocytoma and paraganglioma [PCPG, *n* = 169, (Fishbein et al., 2017)], prostate adenocarcinoma [PRAD, *n* = 397, (Cancer Genome Atlas Research Network, 2015)], sarcoma [SARC, *n* = 247, (Cancer Genome Atlas Research Network, 2017a)], melanoma primary tumors [SKCM, *n* = 72, (Cancer Genome Atlas Network, 2015b)] and melanoma metastases [SKCM-M, *n* = 136], gastric cancer [STAD, *n* = 318, (Cancer Genome Atlas Research Network, 2014a)], thymoma [THYM, *n* = 120, (Radovich et al., 2018)], papillary thyroid cancer [THCA, *n* = 479, (Cancer Genome Atlas Research Network, 2014c)], endometrial carcinoma [UCEC, *n* = 470, (Cancer Genome Atlas Research Network et al., 2013)]. In total, 23 solid tumor types with more than 100 patients per tumor type were included. Ten of them were adenocarcinomas (UCEC, CRC, STAD, BRCA, LIHC, THCA, PRAD, LUAD, PAAD, OV), four were mainly squamous cell carcinomas (HNSC, LUSC, CESC, ESCA) and nine were other tumor types, so neither adeno carcinoma or squamous cell carcinoma (LGG, KIRP, GBM, KIRC, BLCA, SARC, PCPG, SKCM, THYM). Although the total patient number was higher than 100, Germ Cell Tumor (TGCT) was not analyzed because this dataset included a wide variety of tumor differentiation patterns, with less than 100 cases per tumor type. Slides from 7,829 patients from the TCGA archive were all from formalin-fixed paraffin-embedded (FFPE) samples.

### Image Preprocessing

Regions with tumor were manually annotated with QuPath v0.1.2 (Bankhead et al., 2017) by trained observers in every whole slide image (WSI). The non-pathologist observers were initially trained by experienced histopathologists and consulted the histopathologist to resolve difficult cases. Cases were excluded if the image was of poor quality or did not contain any tumor. Subsequently, the tumor regions within whole slide images were tessellated into tiles of 256 × 256 μm^2^ at 0.5 μm per pixel. All data was pre-processed according to the “Aachen Protocol for Deep Learning Histology” (Muti et al., 2020).

### Experimental Design and Preprocessing of Mutation Data

Mutation data of all cases was obtained from www.cbioportal.org, accessed on 05/17/19. We included all genes with a mutation prevalence above 5% in cancer populations. In order to select a set of clinically relevant genes, the target genes were selected based on the prevalence of mutations in the MSK-IMPACT Clinical Sequencing Cohort (MSKCC) and OnkoKB (https://www.oncokb.org/, accessed on 06/12/19). In total, 69 genes were analyzed (Figure 1B): 18 oncogenes, 44 tumor suppressor genes and seven other genes (Supplementary Figure S1). We then ran four different experiments as described as follows. **Experiment #1, “single gene predictability experiment”:** For each mutation in each gene, we manually checked whether it is a likely predicted oncogenic mutation based on OnkoKB, Cancer Hotspots, 3D Hotspot or My Cancer Genome (accessed via cbioportal). Based on this, each patient was assigned a status for each gene: mutated (mutated, mutation clinically relevant), wild type (not mutated, mutation not clinically relevant) or inconclusive (no data). For each genetic alteration (mutation of a given gene), we subsequently trained a Deep Learning system to distinguish mutated from wild type cases, counting inconclusive cases as wild type (WT) in order to only include mutated genes in the analysis. **Experiment #2, “pathway predictability experiment”:** For the analysis of alterations in pathways, the 69 genes were manually assigned to signaling pathways based on a reference publication (Sanchez-Vega et al., 2018). Genes that were not included in the reference publication were manually assigned to pathways based on an additional review of OncoKB (https://www.oncokb.org/), cbioportal (www.cbioportal.org), Gene cards (https://www.genecards.org/, accessed on 06/12/19) and MyCancerGenome (https://www.mycancergenome.org/, accessed on 06/12/19) databases, literature and expert opinion (Supplementary Table S1). In total, 59 genes could be assigned to 12 pathways (Supplementary Figure S1): *MAPK, p53, PI3K, Cell cycle, TGFbeta, Hippo, Notch, FOXA1/ESR1, SWI/SNF complex, Jak-STAT, Wnt, Histone Methylation*. The remaining 10 genes could not be assigned to a particular pathway and grouped as “unknown” pathway. Whenever at least one gene assigned to a particular pathway was found to be mutated, the whole pathway was classified as mutated (“pathway-altered”) in the tumor; whenever none of the genes were mutated, the pathway was labelled as wild type (“not pathway altered”). For each pathway in each tumor type, we then trained the Deep Learning network to distinguish tumors with altered from those with non-altered pathways (Figure 1B). **Experiment #3, “pathway predictability experiment with exclusion of dominant genes”:** In addition, we investigated if the predictability of alterations in the pathway was only driven by alterations in a small set of “dominant” genes. To do this, the prediction experiments of pathway-alterations were repeated for three tumor types (UCEC, STAD and CRC) for three pathways (*p53, MAPK, PI3K*), excluding the following genes: *TP53* in *p53* pathway, *BRAF* and *KRAS* in *MAPK* pathway, and *PIK3CA* and *PTEN* for the *PI3K* pathway. The aim of this experiment was to investigate if the predictability of alterations in pathways is driven by alterations in one or two genes or by alterations in a larger set of genes. **Experiment #4, “allele frequency experiment”:** Lastly, we performed a correlation analysis between the Deep Learning patient scores and allele frequency for the genes *KRAS* and *TP53* genes across all tumor types.

### Deep Learning and Statistics

The general aim of our study was to predict the status of binary targets (single gene mutations or pathway alterations present versus absent) directly from H&E-stained histology image data by Deep Learning. We trained a modified shufflenet for every target as described before (Kather et al., 2020). For each target, the cohort was randomly split into three parts in a stratified way, preserving the proportions of each target level (mutated or wild type). Then, the Deep Learning network was trained in a 3-fold cross-validation approach on the level of patients, ensuring that no image tiles from the same patient were ever part of the training and test set at the same time. Image tiles were only generated from manually annotated tumor regions. Once trained on all tiles in the training set, the network was used to predict the target in each test set tile. Tile-level predictions were subsequently aggregated on the level of patients by simple majority vote and classifier performance was evaluated with a receiver operating curve with 10x bootstrapped 95% pointwise confidence intervals. The primary statistical endpoint was the patient-wise area under the receiver operating curve (AUROC) for each target in each patient cohort. The patient-level prediction scores between patients in the wild type and mutated group for each target were compared by a two-tailed unpaired *t*-test to assess the significance of the separation of groups based on the Deep Learning system. Additionally, for all targets, confusion matrices, F-Score and Matthew correlation coefficient (MCC) with a patient level prediction threshold of 0.5 were calculated and are available in (Supplementary Figure S2 and Supplementary Table S2). Only genes or pathways with at least four patients in each group were analyzed. All source codes are publicly available at https://github.com/jnkather/DeepHistology. A re-implementation of these Matlab codes in Python is available in the histology image analysis package HIA at https://github.com/KatherLab/HIA. All raw histopathology images are available at the TCGA data portal https://portal.gdc.cancer.gov/. All genetic data are available at http://www.cbioportal.org.

## Results

### Prediction of Clinically Relevant Mutations Directly From Histology

**FIGURE 2:**
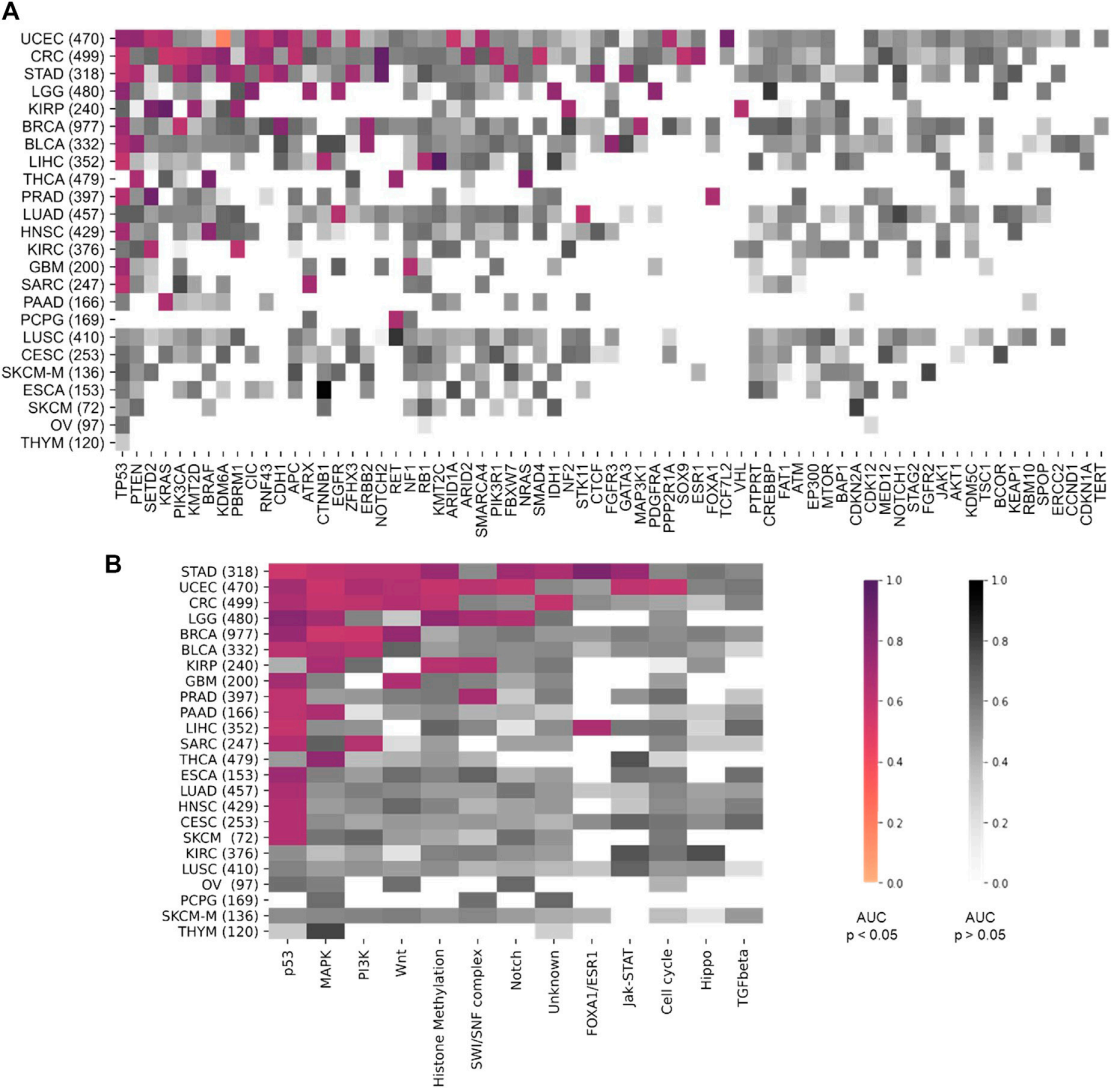
Heatmap comparing the area under the receiver operating curve (AUROC) between the different tumor types. On the y-axis all tumor types are listed and sorted by tumor with most significant results from top to bottom. Number of patients indicated in brackets behind. Pathways are ordered on the x-axis from most (left) to least (right) significant results. AUROC values for **(A)** the twelve pathway analysis and **(B)** for the 69 gene analysis. Coloured values stand for significant detected (*p* > 0.05) pathways and grey for not significantly (*p* > 0.05). TCGA tumor type abbreviations are used (https://gdc.cancer.gov/resources-tcga-users/tcga-code-tables/tcga-study-abbreviations).

First, we performed a comprehensive screen for the predictability of single gene mutations in the tumor types with more than 100 cases in the GDC database (*n* = 23 tumor types, experiment #1). We systematically tested whether the mutation status of the preselected 69 genes with potential clinical relevance with a mutation prevalence above 5% according to the MSKCC and OnkoKB database is directly predictable from histology slides (a list of all genes and prevalence of their mutations in the analyzed data sets is shown in Supplementary Figure S3A and Supplementary Table S3). We found that mutations in 44 out of 69 genes were detectable in one or more tumor types. Most consistently, mutations in *TP53* were predictable in 11 out of 23 cohorts (Figure 2A) with an average AUROC of 0.6812, ranging from 0.597 in hepatocellular carcinoma (LIHC) (0.5320130.677, *p* = 0.035) to 0.787 (0.758–0.823, *p* < 0.001) in low grade glioma (LGG). In addition, in four of the 23 tumor types, alterations in *PTEN, SETD2* and *KRAS* were identified. *PTEN* prediction reached AUROCs of up to 0.773 (0.73–0.799, *p* < 0.001) in UCEC and 0.773 (0.684–0.826, *p* = 0.008) in BLCA. *SETD2* prediction yielded AUROCs of 0.895 (0.827–0.951, *p* = 0.035) in *PRAD*. *KRAS* mutations were predictable with an AUROC of 0.918 (0.844–0.979, *p* < 0.001) in KIRP. The tumor type with consistently highest AUROCs was UCEC, in which AUROCs of 0.764 (0.694–0.8, *p* < 0.001), 0.773 (0.73–0.799, *p* < 0.001), 0.626 (0.527–0.75, *p* = 0.017) and 0.653 (0.595–0.721, *p* < 0.001) were reached for *TP53, PTEN, SETD2* and *KRAS*, respectively. The neural network predicted alterations of twenty genes very well with AUROCs higher than 0.75. Exemplarily seven of these were selected based on expert opinion by a molecular geneticist (NOB), because they were either most clinically relevant, or associated with morphological patterns or prognosis (Table 1). Clinically relevant mutated genes that were chosen were as follows: *FGFR3* in BLCA with an AUROC of 0.78 (0.72–0.822, *p* < 0.001), *IDH1* in LGG with 0.764 (0.735–0.805, *p* < 0.001) and *BRAF* in HNSC with 0.79 (0.739–0.977, *p* = 0.001). Gene mutations associated with morphological patterns were: *BRAF* in THCA 0.86 (0.816–0.886, *p* < 0.001) and *E-Cadherin (CDH1)* in BRCA with 0.81 (0.758–0.849, *p* < 0.001). Prognostically significant mutated genes were: *SETD2* in PRAD with an AUROC of 0.895 (0.827–0.951, *p* = 0.005), *PBRM1* in KIRP with the lowest AUROC 0.752 (0.571–0.939, *p* = 0.006) of these all and lastly *NOTCH2* with the highest AUROC’s in CRC 0.934 (0.893–0.978, *p* < 0.001) and STAD 0.919 (0.846–0.982, *p* < 0.001). All in all, mutations in 25 genes could not be predicted by the neural network in the gene analysis (Figure 2A). Since AUROC is susceptible to different group sizes, we also analyzed the F-Score and MCC (Supplementary Table S2 and Supplementary Figure S2), which showed consistent findings with AUROCs. Among the ten highest F-Scores, *TP53* was found four times. F-scores ranged from 0.846 for *IDH1* in LGG to 0.875 for *TP53* in ESCA and highest MCC correlation of 0.612 for *BRAF* in THCA. In a further analysis, we examined how the F-score, MCC and AUROC changed with different numbers of patients (half *n* = 235, third *n* = 157 and quarter *n* = 117) for the genes *KRAS, PTEN* and *TP53* in the tumor type UCEC (*n* = 470) **(** Supplementary Figures S4A–C). For all values, a decreasing trend was seen with a decreasing number of patients. This effect was strongest for *TP53*.

**TABLE 1:** Top genes result overview. Single gene analysis results with area under the receiver operating curve (AUROC), confidence interval and *p*-Value. Selected genes were very well predicted by the neural network with AUROCs at least above 0.75. **(1–3)** *FGFR3, PBRM1, IDH1* are clinically relevant, **(4–6)** *CDH1, BRAF* is associated with different morphological features and **(7–9)** *SETD2, NOTCH2* have prognostic value.

ID	Tumor type	Gene	AUROC	*p*-Value
1	BLCA	*FGFR3*	0.78 [0.72–0.822]	<0.001
2	LGG	*IDH1*	0.764 [0.735–0.805]	<0.001
3	HNSC	*BRAF*	0.79 [0.739–0.977]	=0.001
4	THCA	*BRAF*	0.86 [0.816–0.886]	<0.001
5	BRCA	*CDH1*	0.81 [0.758–0.849]	<0.001
6	PRAD	*SETD2*	0.895 [0.827–0.951]	=0.005
7	KIRP	*PBRM1*	0.752 [0.571–0.939]	=0.006
8	CRC	*NOTCH2*	0.934 [0.893–0.978]	<0.001
9	STAD	*NOTCH2*	0.919 [0.846–0.982]	<0.001

### Prediction of Pathway Alterations Directly From Histology

**FIGURE 3:**
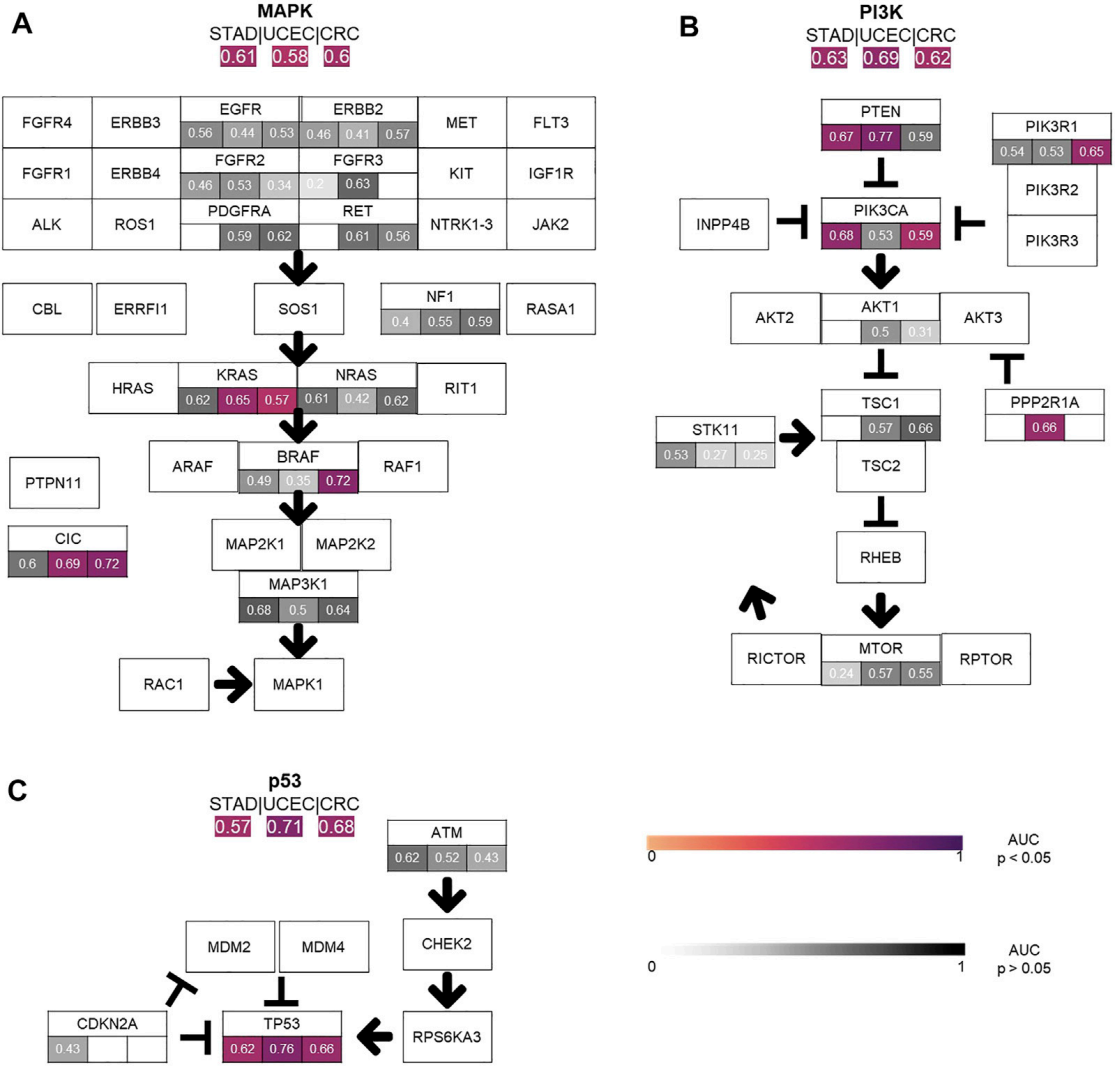
Comparison of the performance of the single gene area under the receiver operating curve (AUROC) vs. pathway AUROC. The top three pathways **(A)** MAPK, **(B)** PI3K, **(C)** TP53 AUROC results for the three top tumor cohorts (STAD, CRC, UCEC) are illustrated. AUROC values are compared between single gene vs. whole pathway. Coloured values stand for significantly detected pathways and grey for not significantly detected (*p* > 0.05).

Next, we tested whether Deep Learning can predict alterations at the level of the selected twelve signaling pathways more easily than the level of individual genes (experiment #2). In this experiment, a pathway in a given tumor was defined to be altered if at least one of the genes in this pathway were mutated (Supplementary Figure S3B). We found that alterations in the pathways *p53, MAPK, PI3K* and *Wnt* were mostly identified by the neural network. The highest number of altered pathways were predictable in gastric cancer (STAD, *n* = 318), endometrial cancer (UCEC, *n* = 470) and colorectal cancer (CRC, *n* = 499) (Figure 2B). In many cases, the detection AUROC values for altered genes were similar to those for altered pathways, e.g., for *TP53* detection 0.66 (0.627–0.718, *p* < 0.001) and the for *p53* pathway detection 0.682 (0.668–0.698, *p* < 0.001) in CRC or for *TP53* detection 0.764 (0.694–0.8, *p* < 0.001) and for *p53* pathway detection 0.71 (0.677–0.738, *p* < 0.001) in UCEC. In summary, the AUROCs for altered pathways were in general lower than for individual altered genes (Figures 2, 3) and training on pathway alterations instead of single gene alterations did not consistently yield a higher performance in the 23 cohorts that we analyzed. Based on these data we hypothesized that predictability of alterations in pathways could be primarily driven by the presence of mutations in one or two genes. To address this, we repeated the analysis for predictability of pathway alterations, but excluded the best predictive genes (experiment #3). In this analysis, alterations in the three pathways *p53, MAPK* and *PI3K* could not be significantly predicted, except in the *MAPK* pathway in the STAD cohort with an AUROC 0.633 (0.609–0.699, *p* = 0.013). The predictability of single gene or pathway alterations showed a positive correlation with the absolute number of mutated cases in a given cohort. In BLCA, the status of the genes *PTEN* (MUT = 12, WT = 398), *ERBB2* (MUT = 39, WT = 371) and *FGFR3* (MUT = 64, WT = 346) were all detected with high AUROCs of 0.773 (0.684–0.826, *p* = 0.008), 0.747 (0.64–0.837, *p* < 0.001) and 0.78 (0.72–0.822, *p* < 0.001), respectively. In the BRCA cohort, the status of the genes *CDH1* (MUT = 106, WT = 871), *TP53* (MUT = 311, WT = 666), *MAP3K1* (MUT = 66, WT = 911), *ERBB2* (MUT = 17, WT = 960) and *PIK3CA* (MUT = 312, WT = 661) were significantly (*p* < 0.05) detected with AUROCs above 0.611. The following tumor types had the highest number of predictable genes, and also highest patient numbers: 470 (UCEC), 499 (CRC) and 318 (STAD), while the lowest predictability was seen in tumor types with 97 (OV), 233 (SKCM primary and metastasis) and 120 (THYM) patients in this cohort. However, this relationship was not absolute as for example in KIRP (240 patients), more single gene alterations and pathway alterations were predictable than in LUSC (410 patients). Therefore, we conclude that patient number in a given cohort does not explain the predictability of mutations alone. While alterations of almost all pathways were detectable in one of the tested tumor types, alterations in the pathways *TGF beta* and *Hippo* were not significantly predictable from histology in any tumor type. However, alterations in the gene *SMAD4* could be predicted with an AUROC of 0.601 (0.524–0.669, *p* = 0.045) and likewise mutations of the *NF2* gene reached an AUROC of 0.701 (0.522–0.834, *p* = 0.029). Furthermore, we hypothesized that the predictability of the histological phenotype of a given alteration would correlate with the allele frequency of mutated genes. To test this, we assessed the correlation between patient-level Deep Learning scores and the allele frequency for the genes *TP53* and *KRAS* in all cohorts. However, this analysis failed to demonstrate a significant correlation (Supplementary Table S4 **, experiment #4**).

### Predictability of Alterations in Different Tumor Types

Having trained Deep Learning systems to detect single gene and pathway alterations in solid tumors, we investigated how tumor types differ in terms of predictability of these alterations. Out of all 23 different tumor types, only in six tumors (*n* = 1 LUSC, *n* = 1 CESC, *n* = 1 SKCM, *n* = 1 ESCA, *n* = 1 OV and *n* = 1 THYM) no mutations were detected. Most altered genes were detected in the cohorts UCEC (15), CRC (15) and STAD (13), all adenocarcinomas. In general, alteration of genes and pathways were identified in nine out of ten adenocarcinoma cohorts, (90%). Three out of the four cohorts of squamous cell carcinomas did not show any significant results. Similar results were seen for the pathway analysis: Most pathway alterations were identified in STAD (9), UCEC (9) and CRC (6). All results are available in Supplementary Tables S5, 6.

## Discussion

For more than a century, histopathological tissue slides stained with H&E have been the gold standard to diagnose solid tumors. In 2018, a seminal study showed that these images are not only a valuable resource for tumor diagnosis, but that genetic alterations in clinically relevant driver genes an be detected by Deep Learning in lung cancer (Coudray et al., 2018). In 2018 to 2021, a number of studies extended these findings to other tumor types and a wide range of genetic alterations (Couture et al., 2018; Sha et al., 2019; Sun et al., 2019; Zhang et al., 2019; Echle et al., 2020a). In particular in 2020, multiple studies have applied supervised Deep Learning for pan-cancer detection of genetic alterations from snap-frozen samples (Fu et al., 2020; Kather et al., 2020; Schmauch et al., 2020) of the TCGA database. While in this previous study, only a subset of all available tumor types was analyzed, we have now extended the assessment of Deep Learning-based detection of pan-cancer genetic alterations to a wider range of tumor types (from 14 to 23) and observed high detection rates for some clinically interesting genes. Additionally, we have evaluated our Deep Learning approach on pathway level in comparison to focussing on single gene alterations, which has not been tested in previous studies to our knowledge.

**FIGURE 4:**
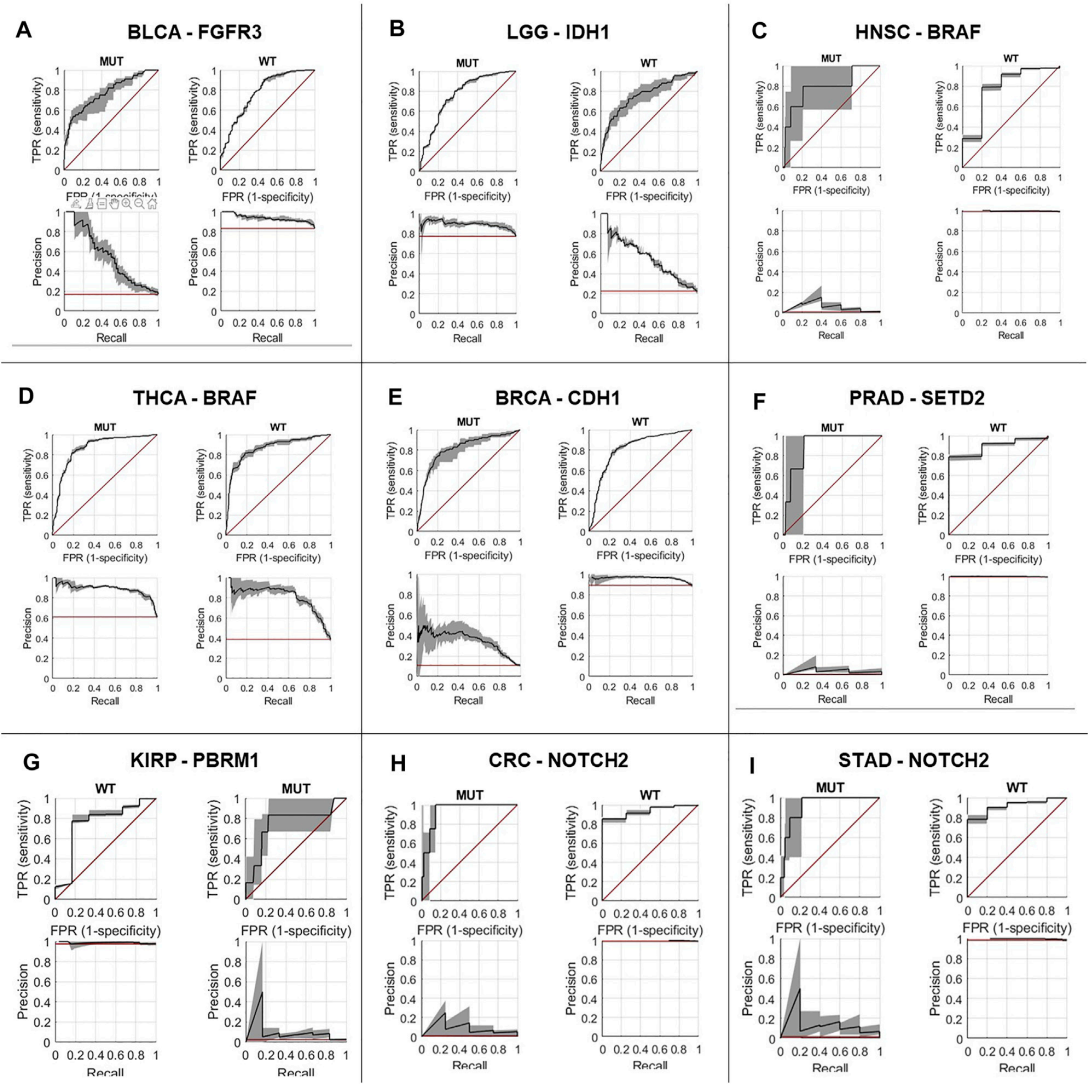
Prediction performance for single gene alterations, representative genes in nine tumor types. Receiver operating curve for: **(A)** *FGFR3* alterations in bladder cancer (BLCA), **(B)** *IDH1* alterations in low grade glioma (LGG), **(C)** *BRAF* alterations in head and neck squamous cell carcinoma (HNSC), **(D)** *BRAF* alterations in thyroid carcinoma (THCA), **(E)** *CDH1* alterations in invasive breast carcinoma (BRCA), **(F)** *SETD2* alterations in prostate adenocarcinoma (PRAD), **(G)** *PBRM1* alterations in renal cell carcinoma (KIRP), **(H)** *NOTCH2* alterations in colorectal adenocarcinoma (CRC), **(I)** *NOTCH2* alterations in stomach adenocarcinoma (STAD), MUT: mutated, WT: wild type

**FIGURE 5:**
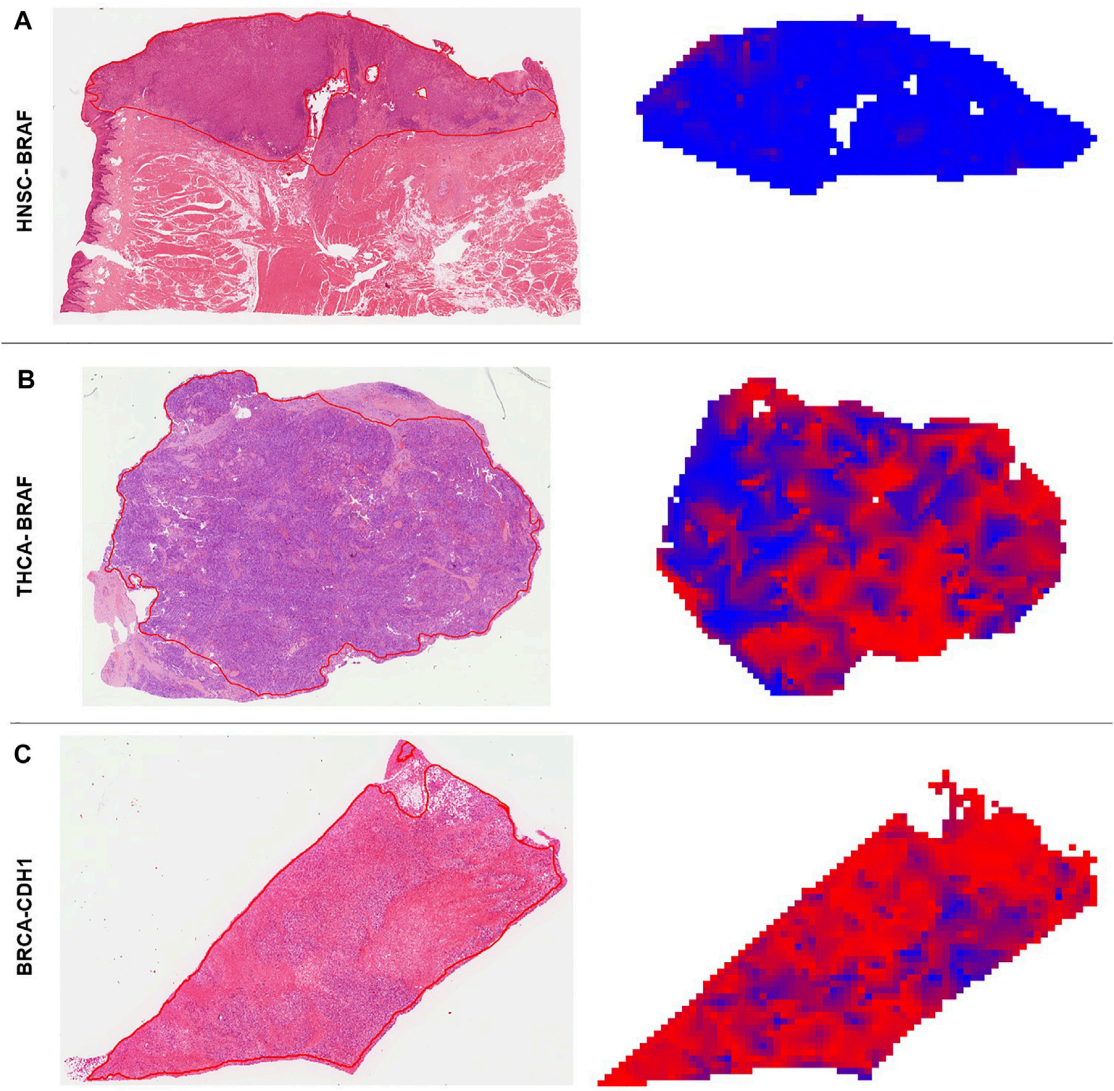
Deep learning predicted heatmaps. Visualization of manually annotated histological slides hematoxylin & eosin (H&E) with corresponding prediction maps for altered genes. Blue areas are wild type (WT) predicted regions and red areas are identified as mutated (MUT) parts by the neural network. **(A)** H&E slide of a *BRAF* WT patient (ID: TCGA-CQ-5333) from the head and neck squamous cell carcinoma (HNSC) cohort. The homogenous blue heatmap is consistent with the wild type status of the patient. **(B)** H&E slide of a *BRAF* mutated patient (ID: TCGA-EL-A3H7) from the thyroid carcinoma (THCA) cohort. The heatmap is more than 50% red, which means the patient was correctly classified as MUT. Intermingled blue areas in tumor regions reflect stroma and artifacts that disturb these areas. **(C)** H&E slide of a *CDH1* mutated patient (ID: TCGA-PE-A5DD) of the breast invasive carcinoma (BRCA) cohort. The prediction heatmap shows that stroma tissue is mostly predicted as WT (blue areas condensed connective tissue) and diffuse invasive-lobular cancer is mostly red.

We found that alterations in single genes were often better predictable from histology than pathway alterations, suggesting that the phenotypic footprint of a pathway is mostly driven by one or two of the genes and that it might be the gene alteration that creates a recognizable pattern, not the pathway alterations itself (Figures 3A–C). The *MAPK* pathway, for example, consists of twelve genes, of whom only three were significantly identified in two cohorts (Figure 3A). This can also be seen in the *PI3K* pathway, where mutations in only two out of eight altered genes were significantly detected in gastric, colorectal and endometrial cancer (Figure 3B). Single gene alteration AUROC’s were similar to those found for altered pathways, e.g., *p53, MAPK* and *PI3K* (Figure 3C). This suggests that a commonly mutated gene might determine the outcome of the pathway analysis in some cases. This hypothesis was verified by our pathway analysis excluding highly predictive genes, as pathway alterations could not be significantly predicted. Interestingly, no gene in the STAD cohort was predicted significantly in the *MAPK* pathway, however the AUROC for the altered pathway as a whole was 0.61 (0.55–0.66, *p* = 0.006) (Figure 3A). Another explanation could also be the higher patient numbers in these tumor cohorts, since this also influences the predictability of alterations in genes and pathways (Figure 3). This was confirmed by a further exemplary analysis in which the AUROC, F-score and MCC decreased with a smaller number of patients in the cohort UCEC **(** Supplementary Figure S4). Direct prediction of mutated single genes from histology images is potentially useful, especially if the alterations have a clinical implication. In general, the neural network could predict several genes—some of which are clinically relevant, associated with morphological pattern or prognostically relevant—very well with AUROCs higher than 0.75. For example, in our study, *FGFR3* mutations could be predicted with an AUROC of 0.78 in bladder cancer (Figure 4A). Since the FDA approved the first targeted-therapy with the *FGFR* inhibitor erdafitinib in advanced muscle invasive bladder cancer (U.S. Food and Drug Administration, 2022), detection of *FGFR3* could identify patients who might benefit from this therapy (Loriot et al., 2019). *IDH1* is an important prognostic marker for brain tumors (Young et al., 2020). In the LGG cohort, 77% (395/512) were *IDH1* mutated, which is associated with a better outcome. *IDH1* could be detected significantly in LGG with an AUROC of 0.764 (Figure 4B). However, in GBM, where only 6% of tumors were mutated, *IDH1* was not significantly detectable. Effectiveness of *IDH* specific enzyme inhibitors in brain tumors are currently tested in clinical trials (Karpel-Massler et al., 2019). The V600E mutation of the *BRAF* kinase gene, which is part of the *MAPK* pathway, plays an important role in tumorigenesis across many types of solid tumors and is in fact a highest level evidence gene of OnkoKB. Although the *MAPK* pathway is altered in many tumor types, *BRAF* mutations are not very common in HNSC (Weber et al., 2003), including the TCGA-HNSC cohort, where only five out of 515 patients showed a *BRAF* alteration. Still, *BRAF* alterations were recognized with a performance of 0.79 in our analysis (Figure 4C and Figure 5A). This makes Deep Learning based identification of subgroups that might benefit from targeted therapy in HNSC conceivable, as specific *BRAF* and *MEK* inhibitors are already an integral part of guideline-directed therapy in other entities. However, interestingly most of the detected mutations in HNSC were indeed non-V600 class II or class III mutations (Yaeger and Corcoran, 2019). In contrast, more than 40% of the thyroid cancers show a BRAF V600 mutation which is associated with the papillary tumor type and found rarely in follicular thyroid cancer type (Nikiforov, 2011) (Figure 5B). In fact, *BRAF* mutational status could be predicted significantly with an AUROC of 0.86 (Figure 4D). Another example is *E-cadherin*, a tumor suppressor gene which is mostly involved in cell adhesion, which is associated with the lobular subtype of breast cancer (Cancer Genome Atlas Network, 2012b) (Figure 5C). In our analysis it was detectable with an AUROC of 0.8 (Figure 4E). Another example is the *PBRM1* gene, which belongs to the *SWI/SNF* chromatin remodelling complex. *PBRM1* is often altered in renal papillary carcinoma, however, recent studies have shown that a *PBRM1* mutation correlates with decreased survival (Ricketts et al., 2018). In our analysis, *PBRM1* was identified with an AUROC of 0.752 (Figure 4F). As *PBRM1* alterations are not very common in papillary renal cell carcinoma, its significance in terms of a potential biomarker remains to be elucidated (Ho et al., 2015; Liu et al., 2020). In KIRP, loss of *PBRM1* has been described to be associated with checkpoint inhibitor resistance (Ho et al., 2015; Liu et al., 2020). Highly predictive image tiles of the five genes mentioned above are collected in Supplementary Figure S5. Other significantly detected prognostic alterations in genes were found in *SETD2* in PRAD with an AUROC of 0.895 (Yuan et al., 2020) and *NOTCH2* in CRC and STAD (Chu et al., 2011) with an high AUROC of 0.934, 0.919 (Figures 4G–I). However, these two genes did not show any relevant pathological features in the top tiles analysis. Analysis of phenotypic footprint of an alteration did not correlate with the allele frequency of mutated genes (Supplementary Table S4).

Based on our overall results, genetic alterations in adenocarcinomas were better predictable than alterations in other tumor types, such as squamous cell carcinomas (Figure 2). This is consistent with previous studies (Schmauch et al., 2020) and leads us to hypothesize that in tumor types with glandular architecture genetic changes might more frequently result in morphological changes and therefore better detectable. Some tumors, predominantly UCEC, CRC and STAD, had more numerous significant findings than others. Interestingly, these are not the tumors with high mutational burden (Kandoth et al., 2013).

In summary, H&E stained tumor images contain subtle morphogenetic information which is detectable by Deep Learning. Our findings correlate with similar results of other Deep Learning analyses and mutational landscape across cancers (Kandoth et al., 2013; Fu et al., 2020; Kather et al., 2020).

### Limitations

Our study has a number of limitations. first and foremost the use of TCGA as our only resource for histopathological whole slide images, which means that validation on additional cohorts is necessary to confirm the results. In addition, a potential confounder in our study is the unequal dataset size for different tumor types in TCGA. It is possible and likely that our study underestimated the number of predictable genes in some tumor types. Especially for small cohorts in this study, future studies should re-analyze the same set of genes in larger cohorts, once such cohorts become available. Finally, while this archive is undoubtedly the most comprehensive multicentric resource available to computational pathology researchers, it has been shown to carry a risk of bias due to the patient selection process in TCGA (Howard et al., 2020). However, a full genetic characterization of thousands of tumor samples like in TCGA is an almost impossible task for academic research groups, which is why TCGA remains very useful and unique to develop and test new computational pathology approaches. Yet, even the genomic characterization in TCGA carries some ambiguity, e.g., due to the presence of non-tumor tissue in sequenced samples as well as different methods for mutation calling. We focussed on single nucleotide variants and small deletions/insertions, and did not take into account fusion genes, copy number changes or expression data. We also relied on a conservative variant classification and therefore might have created a bias regarding the inclusion of “false negative” samples. In future studies, it could be interesting not only to include clinical variant classification data but instead also narrow down the number of included unclassified variants by using prediction algorithms as e.g., BoostDM (https://www.intogen.org/boostdm/search). The most promising candidates for clinical translation should be evaluated in other multicentric image collections obtained via academic consortia. Another limitation of our study is that the tissue slides which we used for our prediction do not necessarily contain the same region that the DNA for genetic characterization has been extracted from. Therefore, it is conceivable that intratumor heterogeneity could dilute our results, potentially leading to a lower performance. Further studies are needed to systematically quantify the impact of intratumor genetic heterogeneity on the inference of genetic alterations from pathology images.

## Data Availability

Publicly available datasets were analyzed in this study. This data can be found here: https://portal.gdc.cancer.gov/.

## Supplementary Material

Supplementary Figure S1 Overview of the genes and pathways arrangement. Following 59 genes were grouped into twelve different cancer pathways. Legend shows characteristics of the genes (orange, oncogenes; blue, tumor suppressor genes; green, both). Information was taken from OnkoKB (https://oncokb.org/).

Supplementary Figure S2 Diagram of F-Score and Matthews Correlation Coefficient vs. AUROC. **(A)** Shown are the F-Score values vs. area under the receiver operating curve (AUROC) for each investigated target of all 23 tumours. **(B)** Shown are the Matthews Correlation Coefficient (MCC) values vs. AUROC for each investigated target of all 23 tumours.

Supplementary Figure S3 Prevalence of mutations in genes and pathways in all tumor types. **(A)** Mutation prevalence in the analyzed data set. **(B)** Prevalence of pathway alterations in the analyzed data set.

Supplementary Figure S4 Diagram AUROC, F-Score and Matthew Correlation Coefficient (MCC) for different patient numbers. **(A)** Changes in area under the receiver operating curve, F-Score and MCC with half, third and fourth patient numbers in tumor type UCEC for target *KRAS*. **(B)** Changes in area under the receiver operating curve, F-Score and MCC with half, third and fourth patient numbers in tumor type UCEC for target *PTEN*. **(C)** Changes in area under the receiver operating curve, F-Score and MCC with half, third and fourth patient numbers in tumor type UCEC for target *TP53*.

Supplementary Figure S5 Highly predictive image tiles selected by deep learning. Comparison of the highly ranked mutated (MUT) and wild type (WT) tiles. This visualization helps to identify morphological changes due to alterations of the tumor. **(A)** *FGFR3* in BLCA: MUT top tiles exhibit more papillary structured parts, whereas WT tiles are more diffuse infiltrative tumor parts. **(B)** *PBRM1* in KIRP: MUT tiles are more solid with rosette-like arrangements, whereas WT tiles have a papillary architecture. **(C)** *IDH1* in LGG: MUT tiles show more glial and fibril appearance. In contrast, WT tiles have a higher nuclear density. **(D)** *CDH1* in BRCA: MUT tiles show the diffusely infiltrating, indian file pattern of lobular-invasive breast cancer, while WT tiles have the trabecular, nodular or tubular architecture of no-special type carcinomas. **(E)** *BRAF* in HNSC: MUT tiles are high grade cancers without any squamoid/cornified elements, whereas in the WT tiles squamous cells and cornification can be found. **(F)** *BRAF* in THCA: MUT of *BRAF* is associated with papillary tumor types and WT tiles show a more organ specific follicular/ colloidal histology.

Supplementary Table S1 Raw results (Microsoft Excel file) of all genes and grouping based on different data sources.

Supplementary Table S2 Confusion matrices, F-Score and Matthews Correlation Coefficient (MCC) for each target, based on a patient-level cutoff of 0.5.

Supplementary Table S3 Mutation prevalences for pathway and gene analysis.

Supplementary Table S4 Correlation analysis of allele frequency with mutation score for *TP53* and *KRAS*.

Supplementary Table S5 Excel File for all AUROC combined Pathway and Genes.

Supplementary Table S6 Prediction results for all tumor types with all statistics. AUROC_avg, average area under the receiver operating curve (AUROC); AUROC_low, lower bound of AUROC confidence interval (CI); AUROC_high, upper bound of AUROC CI; AUCPR_avg, average area under the precision recall curve (AUCPR); AUCPR_low, lower bound of AUCPR CI; AUCPR_high, upper bound of AUCPR CI.; meanCat, mean prediction values for patients in this category; meanOth, mean prediction values for other patients; pVal, p-value for comparison of prediction scores between patients in target category vs. not in target category (according to the ground truth). nPatsTotal, total number of patients (sum of patients in category and not in category).
