# Supplementary figures and images for: Predicting Mutational Status of Driver and Suppressor Genes Directly from Histopathology With Deep Learning: A Systematic Study Across 23 Solid Tumor Types
Source: Front Genet. 2022 Feb 16;12:806386. doi: 10.3389/fgene.2021.806386 (PMC8889144; doi:10.3389/fgene.2021.806386)

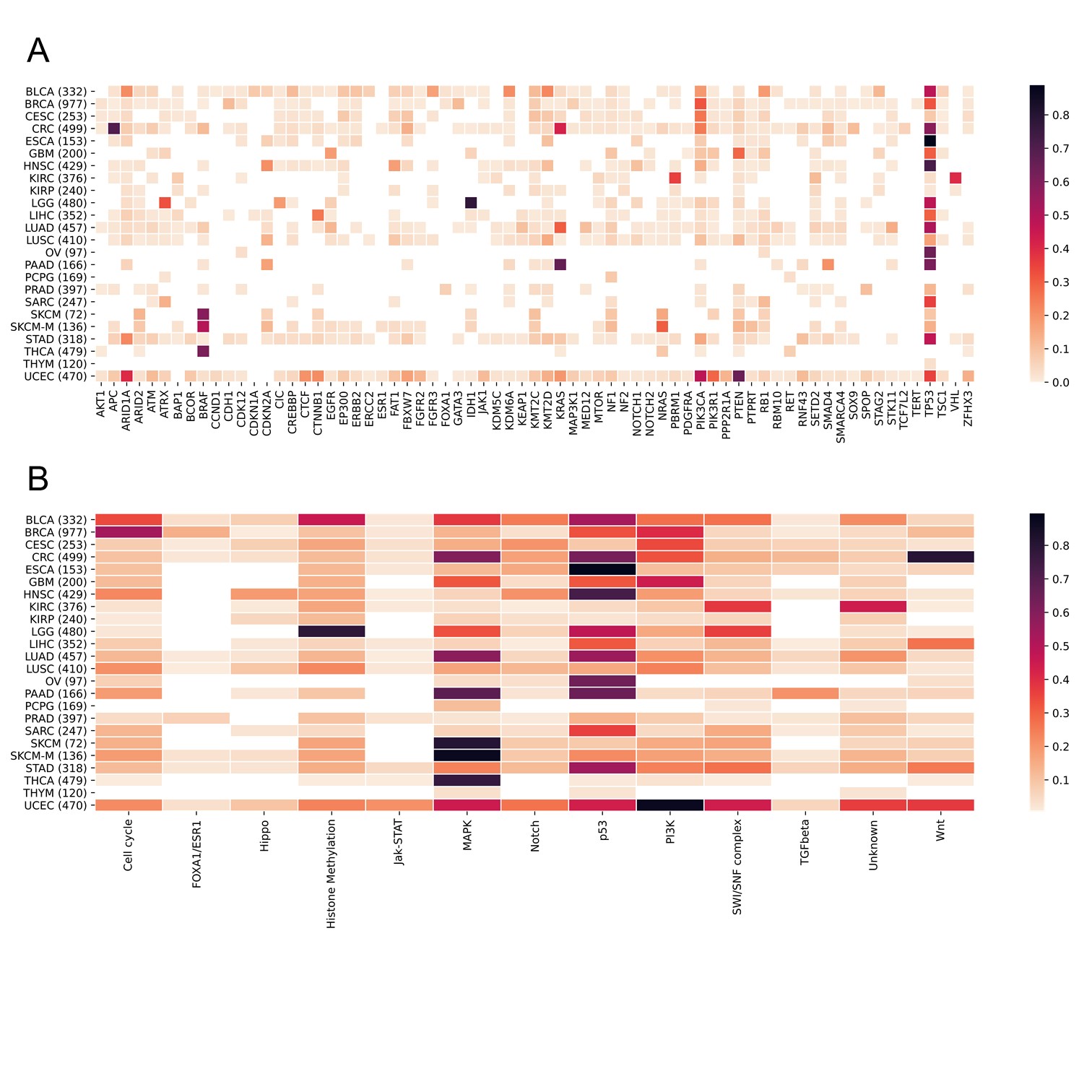

Supplement: Supplementary file 2 [file Image3.JPEG]

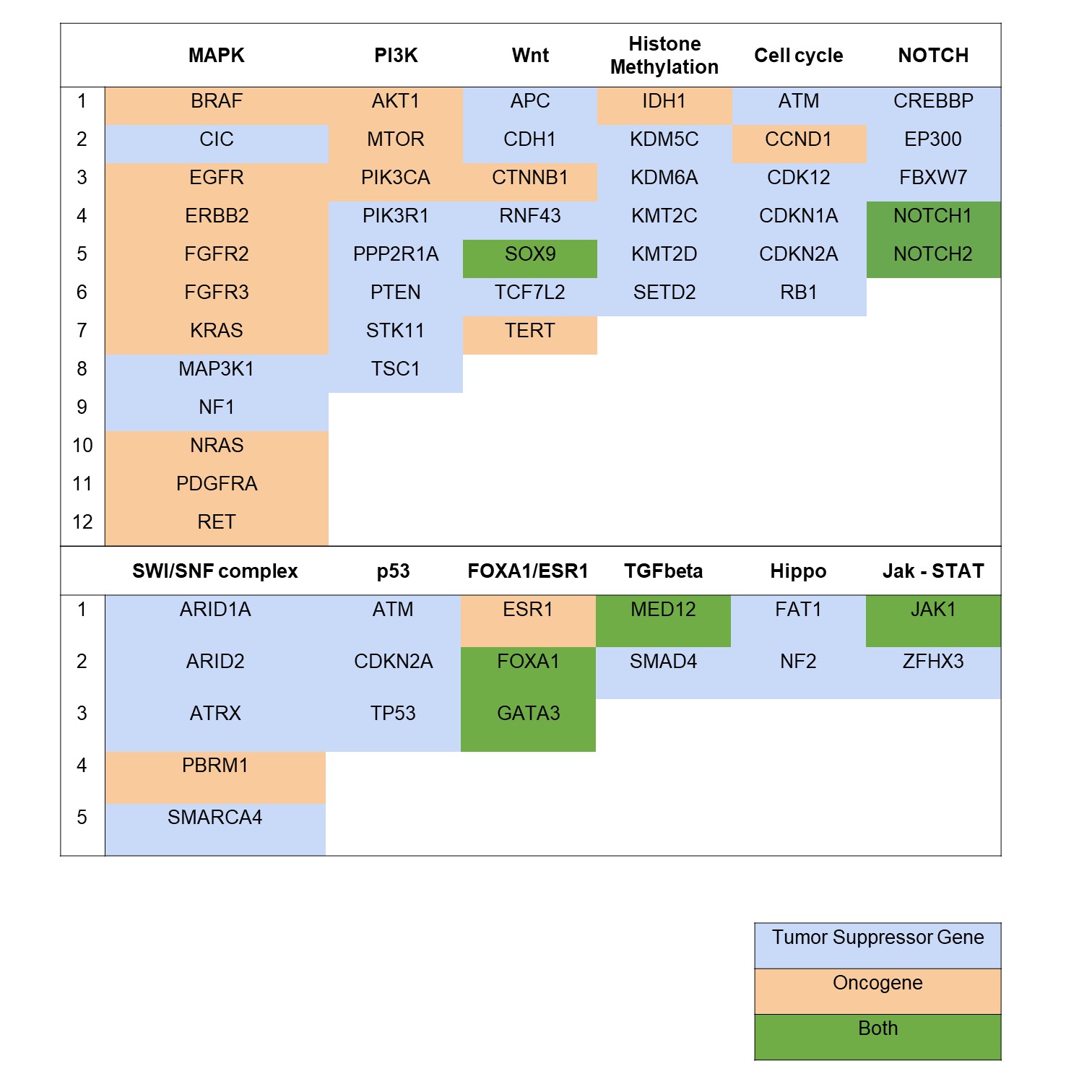

Supplement: Supplementary file 4 [file Image1.JPEG]

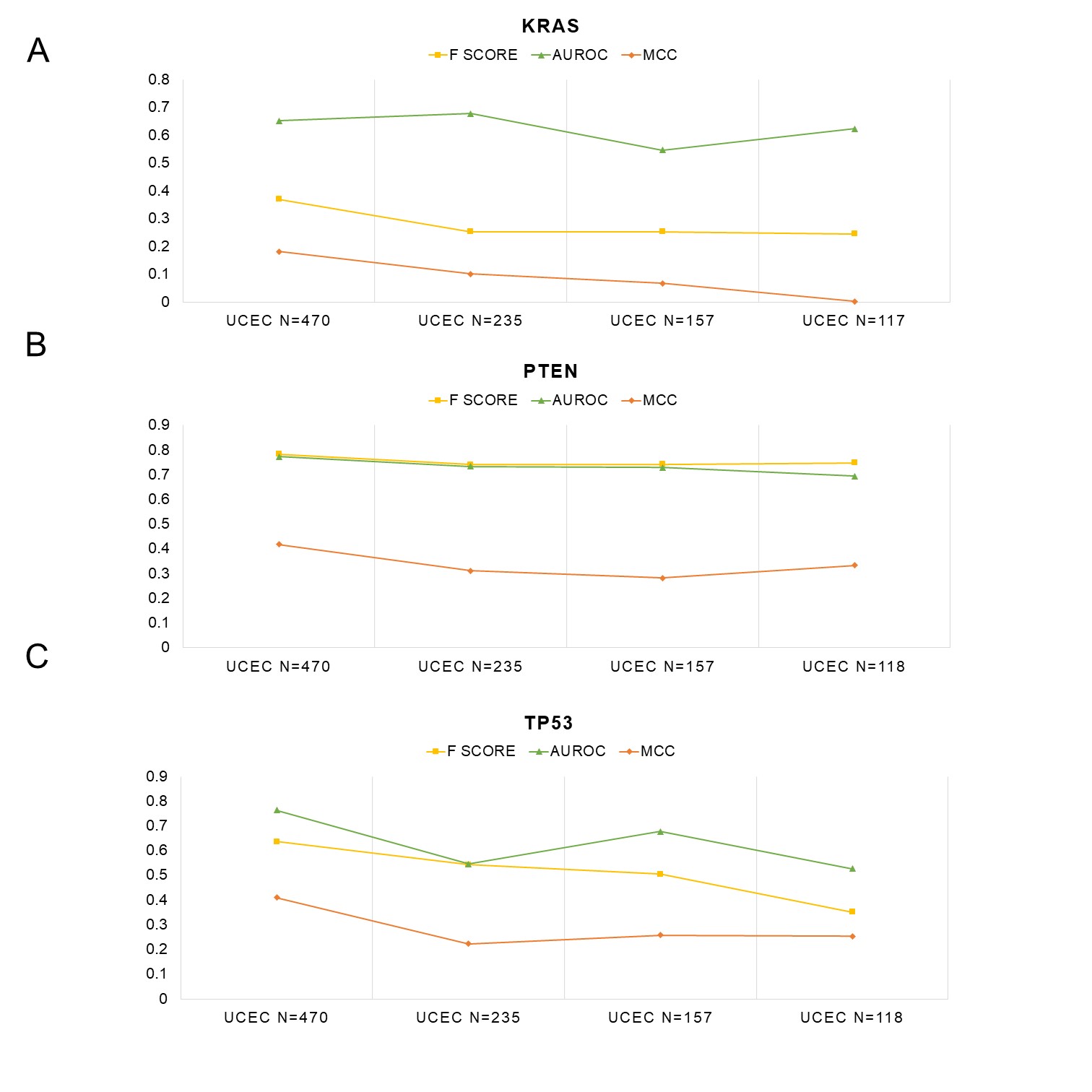

Supplement: Supplementary file 5 [file Image4.JPEG]

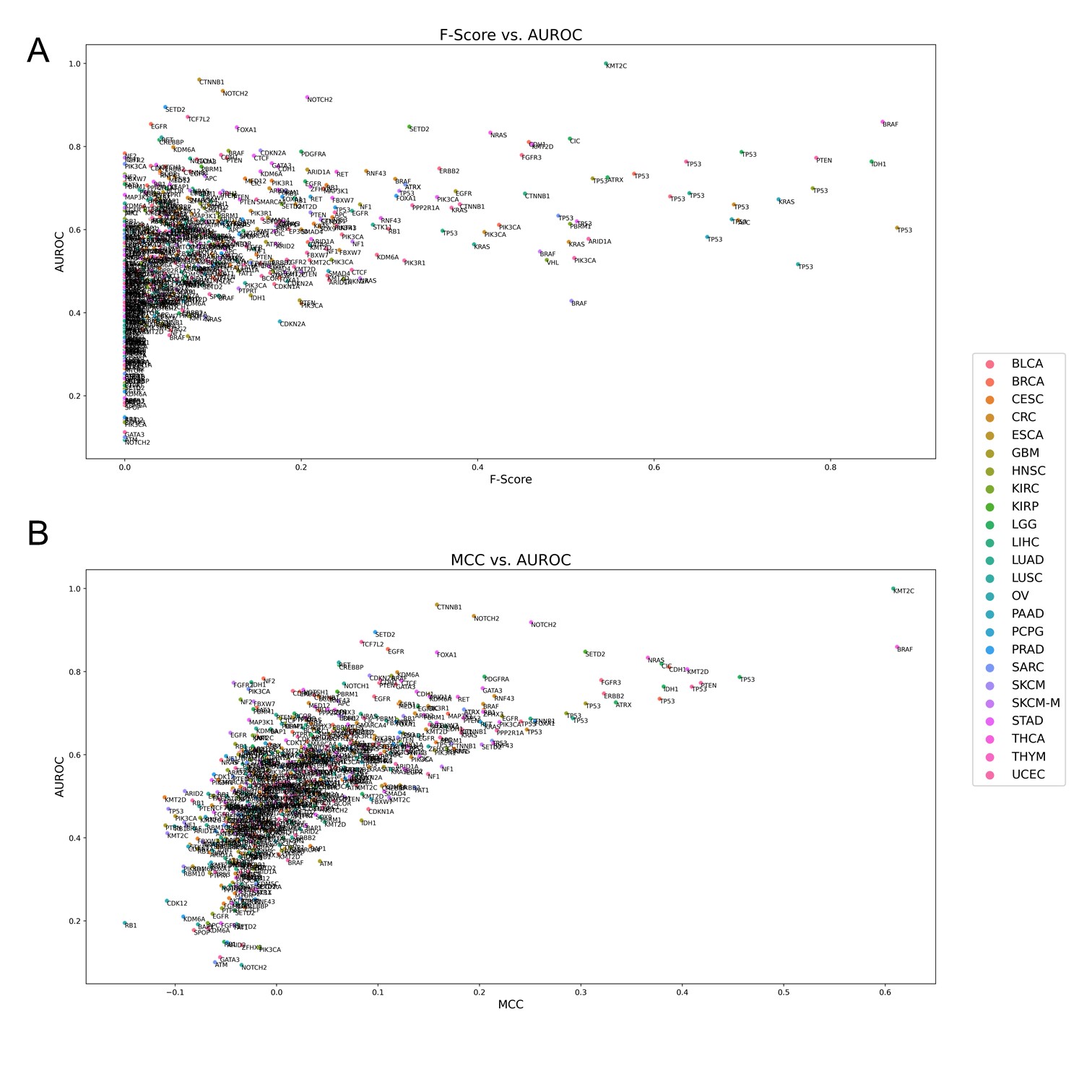

Supplement: Supplementary file 6 [file Image2.JPEG]

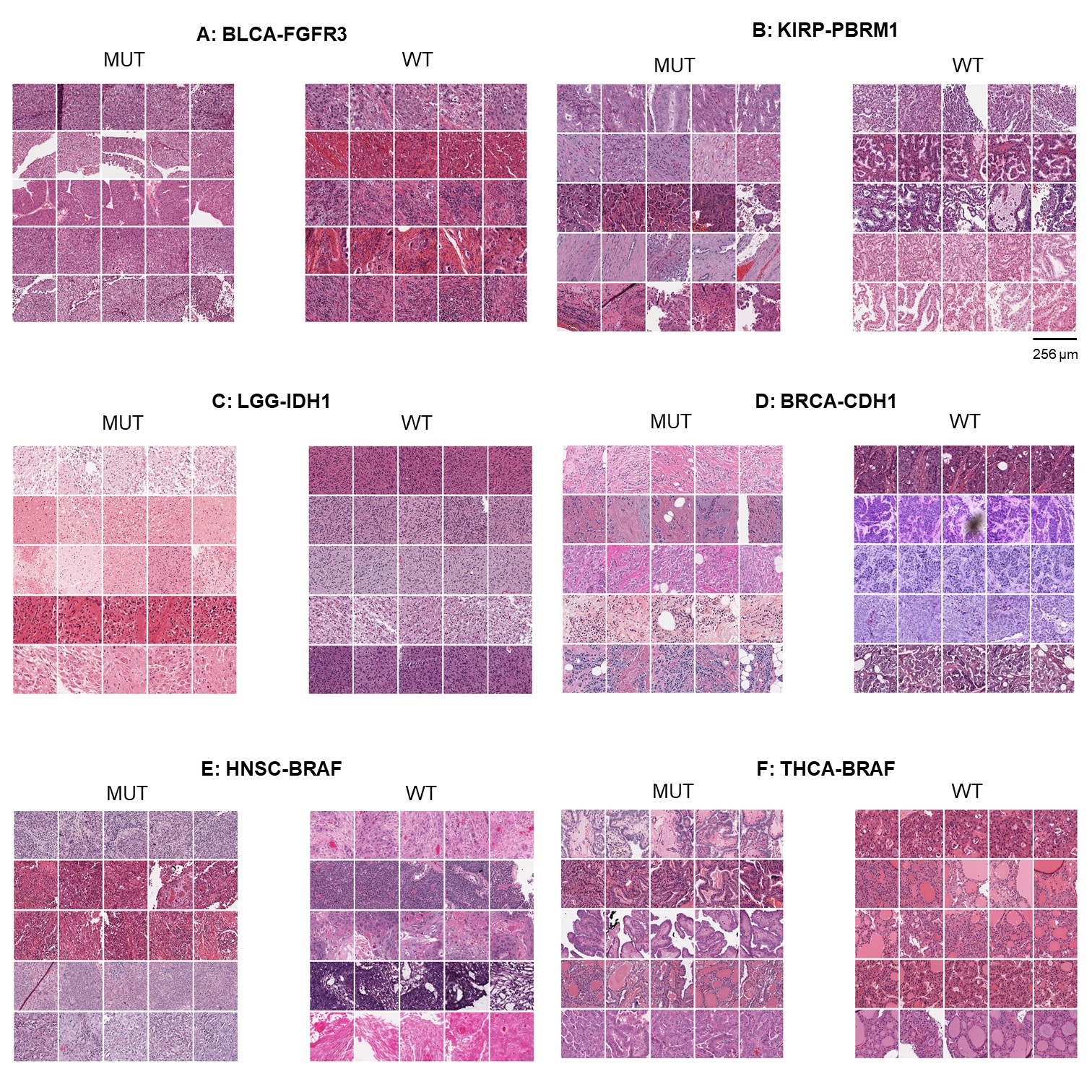

Supplement: Supplementary file 7 [file Image5.JPEG]
